# Prevalence of HDV infection in people living with HIV: Data from a multicenter Italian cohort

**DOI:** 10.3389/fmed.2023.1086012

**Published:** 2023-01-27

**Authors:** Laura Ambra Nicolini, Barbara Menzaghi, Elena Ricci, Emanuele Pontali, Giovanni Cenderello, Giancarlo Orofino, Antonio Cascio, Giovanni Francesco Pellicanò, Laura Valsecchi, Chiara Molteni, Francesca Vichi, Paolo Bonfanti, Antonio Di Biagio

**Affiliations:** ^1^Unit of Infectious Diseases, IRCCS Ospedale Policlinico San Martino, Genoa, Italy; ^2^Unit of Infectious Diseases, ASST Della Valle Olona—Busto Arsizio (VA), Busto Arsizio, Italy; ^3^Fondazione ASIA Onlus, Milan, Italy; ^4^Department of Infectious Diseases, Galliera Hospital, Genoa, Italy; ^5^Department of Infectious Diseases, Sanremo Hospital, Sanremo, Italy; ^6^Division I of Infectious and Tropical Diseases, ASL Città di Torino, Torino, Italy; ^7^Unit of Infectious Diseases, Department of Health Promotion, Mother and Child Care, Internal Medicine and Medical Specialties, University of Palermo, Palermo, Italy; ^8^Unit of Infectious Diseases, Department of Human Pathology of the Adult and the Developmental Age “G. Barresi”, The University of Messina, Messina, Italy; ^9^1st Department of Infectious Diseases, ASST Fatebenefratelli Sacco, Milan, Italy; ^10^Unit of Infectious Diseases, A. Manzoni Hospital, Lecco, Italy; ^11^Department of Infectious Diseases, SOC 1 USLCENTRO Firenze, Santa Maria Annunziata Hospital, Florence, Italy; ^12^Infectious Diseases Unit, Fondazione IRCCS San Gerardo dei Tintori, Monza, Italy; ^13^University of Milano-Bicocca, Milan, Italy; ^14^Department of Health Science (Dissal), University of Genoa, Genoa, Italy

**Keywords:** HIV, bulevirtide, treatment, HDV, prevalence

## Abstract

**Objectives:**

The development of novel antiviral agents active against Hepatitis Delta Virus (HDV) might change the natural history of chronic infection, reducing the risk for end-stage liver disease. People living with HIV (PWH) are at risk for bloodborne pathogens infection, but limited data on epidemiology of HDV infection is available in this setting. The aim of this study was to investigate HDV prevalence and attitude toward HDV testing and treatment in infectious diseases centers.

**Methods:**

A cross sectional survey was performed among centers participating in the CISAI (Coordinamento Italiano per lo Studio dell’Allergia in Infezione da HIV) Group. The survey addressed anti-HDV prevalence and HDV-RNA detectability rates in PWH as well as perceived obstacles to treatment.

**Results:**

Overall, responses from ten sites were collected. Among participating centers, 316 PWH with HBV chronic infection are currently followed. Of them, 15.2% had positive anti-HDV antibodies, while 13.9% were not tested yet. Overall, 17% of anti-HDV positive PWH tested at least once for HDV-RNA had active HDV infection, and 71% of them had advanced liver disease. Most infectious diseases centers intend to treat locally HDV infection with upcoming anti-HDV drugs, but some concerns exist regarding treatment schedule.

**Discussion:**

HDV testing needs to be implemented in PWH. At present, few patients followed in the CISAI centers seem to be candidate to receive new direct active anti-HDV agents, but repeated HDV-RNA measures could change this proportion.

## Background

According to a recent meta-analysis, approximately 12 million people worldwide live with hepatitis Delta virus (HDV) infection, and up to 64% of anti-HDV positive people have chronic HDV replication (HDV-RNA) (1). HDV is a defective virus that requires hepatitis B virus (HBV) surface antigen (HBsAg) to cause liver infection and disease. Chronic HDV infection poses patients at risk for liver cirrhosis, clinical decompensation, and development of hepatocellular carcinoma (HCC) (2). Thus, HDV infection is a major health problem that needs to be addressed in order to reduce liver-related mortality.

The risk of developing HBV and HDV infection is higher in people living with HIV (PWH), and PWH are at higher risk of developing chronic HBV infection (3, 4). Moreover, PWH coinfected with HBV experience more frequently cirrhosis and its complication than people living without HIV (5). Additionally, data from the Swiss HIV cohort study highlight that HDV infection is strongly associated with overall and liver related death as well as with the occurrence of HCC in PWH (4). Reasons for the exceeding risk are still unclear, but it has been supposed that impaired immune surveillance due to HIV infection could promote the development of HCC (6).

Notably, HDV prevalence has changed over time, and it is difficult to understand the current extent of the problem. In Italy, the proportions of anti-HDV positivity in PWH dropped from 28% in 1997 to 4% in 2011, then it rebounded to 8% in the period 2012–2015 (7).

Persistent HDV replication is the only identified predictor of liver-related events, including cirrhosis and HCC, in anti-HDV positive people. Additionally, levels of HDV-RNA seem to predict liver disease progression (8, 9), while persistent HDV-RNA suppression following treatment results in reduced liver-related mortality and increased cumulative event free survival (10, 11). Although the clinical significance of HDV-RNA is clear, data on HDV prevalence usually focuses on anti-HDV seroprevalence, while a few studies reported on HDV viraemic infection.

The aim of this survey was to investigate the prevalence of anti-HDV and replicative HDV infection as well as the attitude toward HDV treatment in PWH, in a large Italian HIV network.

## Methods

A cross sectional survey was performed among centers participating in the CISAI (Coordinamento Italiano per lo Studio dell’Allergia in Infezione da HIV) Group, a collaborative group of Italian HIV clinics (12). The survey included 10 questions and was advertised by email to the HIV clinic directors. Following first advise, a remind was sent a few weeks later. Participation was voluntary and not compensated. Participating sites provided raw data that were subsequently elaborated. Although participation was not anonymous, no information on characteristics of participating sites was asked. Results of the survey were discussed during the CISAI annual meeting in May 2022.

In the survey, the first questions addressed the prevalence of anti-HDV and HDV-RNA in PWH with detectable HBsAg, the proportion of untested PWH, and features of HDV-related liver disease (i.e., grade of liver fibrosis and presence of liver cirrhosis).

According to the study coordinating center procedures, a cut-off of 14 kPa at transient elastography was used for the diagnosis of cirrhosis. Metavir F3 fibrosis was defines as liver stiffness between 10.1 and 14, while Metavir F2 for liver stiffness between 8 and 10 kPa (13).

Previous treatment with interferon was also investigated. Regarding upcoming treatment options for HDV, we asked whether eligible patients would receive treatment on-site or they would be addressed to an hepatologist referral center. Finally, a close ended question was used to investigate potential issues related to HDV treatment.

## Results

**FIGURE 1:**
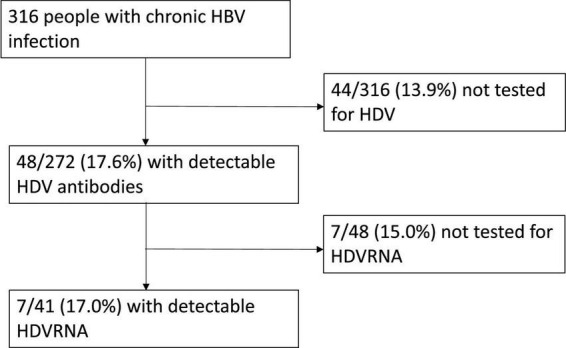
Flow chart of the study population.

Ten centers answered the questionnaire. Overall, 316 PWH with HBV chronic infection referred to the participating centers (Figure 1). Of them, 48 (15.2%) had detectable anti-HDV. Of the remaining patients, 44 (13.9%) were not tested for anti-HDV antibodies. A *post hoc* power analysis, performed using the OpenEpi software (14), revealed that our sample size allowed a 99% confidence level that the true anti-HDV prevalence in our cohort is between 14 and 16%.

Among anti-HDV positive patients, 15% (7/48) had never been tested for HDV-RNA. Of tested patients, 7 (17%) had detectable HDV-RNA and were thus regarded as having active HDV co-infection.

Of PWH with active HDV, liver elastography was available for 6 patients and revealed liver cirrhosis in 2 cases, who also present with laboratory and clinical signs of liver cirrhosis, Metavir F3 in 2 and Metavir F2 liver fibrosis in 2 cases. One additional patient received liver transplantation for HCC; after transplant, he did not experience HBV reactivation so far. All data on liver fibrosis were collected according to liver stiffness at transient elastography and were referred to March 2022, except for one patient who received liver elastography in June 2022 as she was diagnosed with HBV/HDV infection at the beginning of the SARS-CoV-2 pandemic. In the meanwhile, she experienced oropharyngeal carcinoma requiring combined surgery, chemotherapy and radiotherapy. Thus, elastography was postponed in order to limit the number of hospital accesses; aspartate aminotransferase (AST) to Platelet Ratio Index (APRI) and Fibrosis-4 (FIB-4) indexes ruled out liver cirrhosis. Overall, 3 (42.9%) patients with active HDV had previously been treated with interferon without HDV eradication.

Regarding the attitude toward upcoming treatment options, all but one centers responded that active HDV co-infection would be treated on-site once new drugs become available. The last center answered that they usually address their patients with hepatic issues to a gastroenterology referral center for chronic liver hepatitis. Reported potential barriers to HDV treatment initiation were deemed: the need to increase frequency of medical visits and blood tests (*n* = 1); the subcutaneous route of administration (*n* = 2), the unclear length of treatment schedule (*n* = 1), and the potential risk for drug-to-drug interactions (DDI) (*n* = 1).

## Discussion

In the present study, we report a seroprevalence rate of HDV infection among PWH HBsAg carriers of 15.2%, while the 13.9% is still waiting for anti-HDV testing. In countries without a generalized HIV epidemic, an epidemiological association between HDV and HIV infection has been reported, probably related to the shared transmission routes (1). HDV prevalence rate we found was consistent with estimated prevalence in HBsAg-positive populations from hepatology clinic in Europe (1), but it was slightly different from those reported by the Swiss and Italian cohorts up to 2015 (4, 8, 15). Indeed, HDV prevalence was 18% in HBsAg positive PWH enrolled between 1988 and 2014 in the Swiss HIV cohort study (4). The ICONA foundation reported an 8% HDV seroprevalence rate in the period 2012–2015 (7). By contrast, a multicentre study in Northern Italy found that approximately one third of PWH seen at one of the participating centers in 2010 had positive HDV serology (13). Of note, our data may partially overlap those from the ICONA and from the Northern Italy, as some CISAI centers also participate in these cohorts. However, our data are updated to 2022 and thus provide a picture of the current epidemiology of HDV infection in PWH.

The proportion of patients untested for anti-HDV is lower than previously reported in the Italian cohorts (7, 16). This data could reflect an increasing attitude toward anti-HDV testing in clinical practice, that might be related to the upcoming availability of anti-HDV drugs. Indeed, up to 2020, no HDV direct-acting antiviral agent was available. Pegylated Interferon was the only drug approved by the European Medicines Agency (EMA), although it did not receive the Food and Drug Administration approval (16). Unfortunately, treatment with pegylated interferon was limited by low efficacy rates, high risk for adverse events and possibility of late relapse of HDV infection (17–19). Recently, new molecules targeting host factors have been developed (20). Among them, bulevirtide is an entry-inhibitor that received conditional marketing authorization by EMA in 2020, based on two small phase II studies (16). Clinical trials and real-word experiences showed that bulevirtide reduced HDV-RNA and normalized alanine aminotransferase levels. However, in clinical trials the treatment duration was limited to 24 weeks and off-treatment virological response was not reported (21, 22). Additionally, clinical trials focused on viraemic individuals and used the combination of > 2log decline in viral load and normalization of alanine aminotransferase, in spite of virological suppression, as surrogate marker for efficacy (21, 22). Further efforts are needed to ensure that all PWH with ongoing HBV infection receive HDV screening, according to international guidelines.

The rate of replicative HDV infection we found was as low as 17%. According to the literature, HDV-RNA detectability rate widely vary (4, 23, 24). Notably, our survey did not focus on viral nuclear extraction protocols and type of assay used to assess presence of HDV-RNA. Additionally, we did not evaluate whether PWH received single or repeated testing for HDV-RNA. Thus, we could not argue on the possibility of false negative results.

The performance of different techniques for the assessment of liver fibrosis in HDV infected patients has not been well-studied so far. While liver biopsy is historically considered the gold standard for disease staging, several non-invasive fibrosis tests have shown to be accurate in evaluating the presence of significant fibrosis and liver cirrhosis in HBV and HCV chronic infection (25). However, validation of these tests in the setting of chronic HDV is still pending. Novel tests recently studied in the setting of HDV are the Delta Fibrosis Score and the D4FS, that have shown promising results in the assessment of advanced liver fibrosis and cirrhosis, respectively (25). Although ideal cut-offs of liver stiffness for staging of liver fibrosis in HDV infected patient with transient elastography are not available, we asked centers to report on liver fibrosis according to liver stiffness, as it is largely used in the setting of HBV infection and easy to report. However, the possibility that liver disease staging with these cut-offs could not be accurate should be acknowledged.

Both hepatologists and infectious diseases specialists usually manage patients with chronic viral hepatitis. However, depending on local and regional organization, patients may be sent to referral centers. As we aimed at investigating HDV in PWH, we conducted the survey among infectious diseases specialists. Notably, among participating centers only one reported that individuals eligible to upcoming HDV treatment would not be treated locally. Concerns regarding treatment included the route of administration and uncertainty regarding treatment duration as well as the need to increase controls despite the recent SARS-CoV-2 pandemics. At present, data on the optimal treatment duration and post-treatment efficacy using bulevirtide are pending (26, 27). Results from phase 3 clinical trials should be soon available and might be helpful in order to address these concerns. Regarding potential DDIs, bulevirtide is a CYP3A4 inhibitor, thus it likely presents some risk. However, as limited information are available, further studies are needed (28).

In summary, despite 15.2% anti-HDV prevalence, we found that 17% of anti-HDV positive PWH harbored active HDV infection and were thus eligible for treatment with bulevirtide and/or other anti-HDV drugs currently under development. HDV chronic infection is difficult to treat, and no standardized screening strategies have been implemented in Central Europe so far (29). Given that new drugs are on the horizon, implementing screening strategies for HDV infection and HDV-RNA testing is pivotal, especially in populations at high risk for HDV infection.

## Data availability statement

The raw data supporting the conclusions of this article will be made available by the authors, without undue reservation.

## Ethics statement

The studies involving human participants were reviewed and approved by Local Ethical Committees participating in the CISAI cohort. Indeed, CISAI supports a prospective, observational, multi-center study created to assess the incidence of adverse events in patients receiving new antiretroviral drugs in clinical practice. It is an online pharmacovigilance program involving 22 Italian Infectious Disease Departments. The coordinating center is ASST Fatebenefratelli Sacco-Milan, Italy. The Project has an Internet site (http://www.cisai.info). The survey of the present study was addressed to the site directors of the participating centers. Patients evaluated in the survey were already enrolled in the project. Participation in the observational multi-center study requires a signed informed consent. The patients/participants provided their written informed consent to participate in this study.

## Author contributions

LN designed the study and wrote the manuscript. AD, BM, and PB supervised the findings of this work. ER performed the computations and worked out the technical details. EP, GC, and GO worked out the clinical aspects of patients with HDV infection. AC, GP, LV, CM, and FV contributed to the interpretation of the results. All authors discussed the results, contributed to the final manuscript, and approved the submitted version.
